# Buprenorphine therapy in the setting of induced opioid withdrawal from oral naltrexone: a case report

**DOI:** 10.1186/s12954-020-00417-9

**Published:** 2020-10-07

**Authors:** Laura M. Szczesniak, Vincent J. Calleo, Ross W. Sullivan

**Affiliations:** 1 Department of Pharmacology, Upstate Medical University, 750 E Adams St, Syracuse, NY 13210 USA; 2 Department of Emergency Medicine, Upstate Medical University, 750 E Adams St, Syracuse, NY 13210 USA

**Keywords:** Buprenorphine, Opioid use disorder, Overdose, Substance use treatment, Precipitated withdrawal, Naltrexone

## Abstract

**Background:**

Patients with opioid use disorder (OUD) frequently present to the emergency department for acute treatment of overdose and withdrawal.

**Case presentation:**

A 29-year-old male presented to the emergency room after intravenous heroin use followed by accidental ingestion of naltrexone. He was treated with buprenorphine with significant improvement in his Clinical Opioid Withdrawal Score, from moderately severe to mild withdrawal symptoms within a few hours.

**Conclusion:**

Buprenorphine and minimal supportive care can be used to treat acute withdrawal precipitated by oral naltrexone in patients with OUD.

## Background

Opioid use disorder (OUD) is a public health emergency, with two out of three overdose deaths in 2018 involving an opioid [1]. In most cases, the Emergency Department (ED) is the primary location for acute treatment of these patients. If proper care is not provided to patients with OUD, they remain at a high risk for morbidity and mortality related to drug abuse.

The increased usage of the opioid antagonist naloxone has played a vital role in treatment of opioid overdose, but its use can lead to precipitated withdrawal, which is a rapid and intense onset of withdrawal symptoms. Several case studies have recently addressed the issue of precipitated withdrawal by treatment with buprenorphine [2–4], a partial opioid agonist that is traditionally used to treat symptoms of long-term withdrawal [5]. Patients in these studies were given buprenorphine after naloxone administration in order to alleviate acute symptoms, which resulted in a reduction in their Clinical Opioid Withdrawal Scores (COWS) [6].

Naltrexone is another opioid antagonist commonly used as abstinence in patients with opioid or alcohol dependence. Ingestion of naltrexone with measurable levels of opioids in the body can precipitate acute withdrawal, but withdrawal symptoms often appear more severe than usual [7]. One recent study found that elective naltrexone-induced withdrawal could be treated with buprenorphine in order to avoid methadone tapering and hasten treatment [8]. To the best of the authors’ knowledge, this is the first case of an accidental oral naltrexone ingestion after intravenous (IV) heroin use that was successfully treated by oral buprenorphine.

### Case presentation

A 29-year-old male with a history of IV drug use presented to the ED with severe abdominal pain and vomiting. He reported injecting heroin in the morning, and approximately 10 min later ingested 50 mg naltrexone after confusing it with another medication. The patient was driving when he had a sudden onset of severe abdominal pain, approximately 1 h after naltrexone ingestion. After continuous 10/10 abdominal pain along with several episodes of emesis and hallucinations, he pulled his car over to the side of the road and was eventually brought to the ED by ambulance, arriving approximately 2.5 h after the naltrexone ingestion. On physical exam, he appeared distressed, diaphoretic, and agitated, with pressured speech. The CBC and BMP were within normal limits, except for decreased potassium (3.1 mmol/L) and increased glucose (163 mg/dL). VBG was significant for alkalosis (pH 7.56), hypocapnia (30 mmHg), decreased vSO_2_ (55%) and lactic acidosis (4.8 mmol/L).

**Fig. 1:**
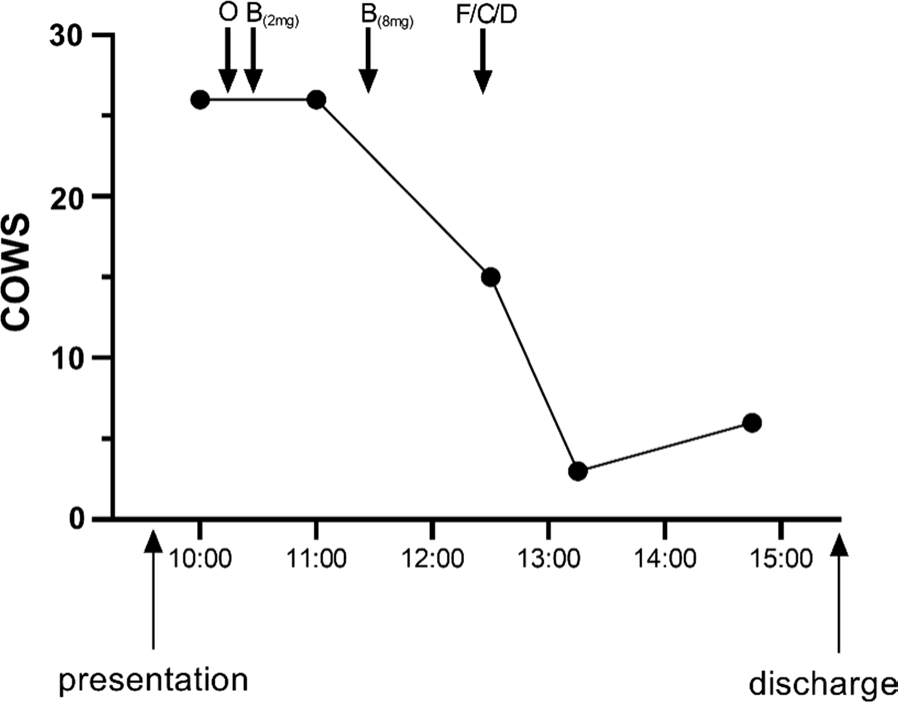
Patient COWS during ED admission, with all treatments shown at the indicated times. O = ondansetron; B = buprenorphine; F/C/D = famotidine/clonidine/diazepam

The patient’s COWS was 26 on initial presentation (Table 1), indicating moderately severe opioid withdrawal [6]. He received 4 mg of IV ondansetron for severe nausea and vomiting, and the decision was made to treat remaining symptoms with buprenorphine. COWS for this patient and the drug administration timeline is shown in Fig. 1. Briefly, 2 mg of oral buprenorphine was administered, followed by 8 mg approximately 1 h after the first dose after little change in COWS was observed. Over the next 90 min, there was significant improvement in the COWS from 26 to 15. At that time, the patient was also administered IV famotidine, clonidine and diazepam. The COWS continued to improve, and within 3 h of presentation, the patient demonstrated only mild withdrawal symptoms. The last two recorded COWS were 3 and 6, respectively. He was discharged with instructions to follow up with the addiction center at this institution. Since presentation, patient has continued IV drug use, but is being followed by our hospital addiction services.

**Table 1:** Patient COWS by individual category [6]

Time	1000	1100	1230	1315	1445
Resting pulse rate	0	1	0	0	1
Sweating	2	2	2	1	1
Restlessness	3	3	3	0	0
Pupil size	2	2	1	1	1
Bone or joint aches	2	2	1	1	1
Runny nose or tearing	2	2	0	0	0
GI upset	5	3	3	0	0
Tremor	2	1	0	0	0
Yawning	1	1	0	0	1
Anxiety or irritability	2	4	2	0	1
Piloerection (“gooseflesh skin”)	5	5	3	0	0
Score	26	26	15	3	6

## Discussion and conclusions

Due to its unique properties as a partial mu agonist, buprenorphine is widely used as a treatment in the setting of long-term opioid withdrawal and OUD in order to deter patients from illicit opioid use or opioid abuse. Buprenorphine induction therapy is also increasingly being considered in the ED rather than through drug rehabilitation clinics due to the current public health crisis surrounding OUD [5]. While buprenorphine activates the mu receptor, it produces significantly less euphoria compared to full mu receptor agonists, such as morphine. However, buprenorphine binds to opioid receptors with a relatively high affinity, often displacing other opioids present in the body. Additionally, buprenorphine alone does not normally cause significant hypoventilation or sedation in adults, making its use in OUD treatment relatively safe and reduces the risk of overdose related to buprenorphine administration.

This patient was initially treated with 2 mg buprenorphine and closely observed due to the severity of withdrawal symptoms. When a repeat COWS indicated that withdrawal symptoms remained moderately severe, we chose to treat with another 8 mg buprenorphine. Without adjunct medications, this patient’s COWS improved from 26 to 15 within 90 min, and improved further to a score of 3 over the next 45 min (Fig. 1). It is important to note that famotidine, clonidine and diazepam were administered during this time, but we do not believe that these medications solely contributed to a further decline to a COWS of 3. GI upset and piloerection (Table 1) completely resolved within the 45 min of receiving these adjuncts, which most likely better reflects buprenorphine action.

We show that buprenorphine along with as-needed supportive care can be used to successfully treat acute precipitated withdrawal after accidental administration of an opioid antagonist, such as naltrexone. The relatively rapid induction (~ 30 min) and long half-life of buprenorphine (~ 37 h) makes its use ideal because it will maintain therapeutic levels for prevention of withdrawal symptoms. Although buprenorphine may cause initial worsening of withdrawal symptoms or COWS score in certain settings due to increased affinity of mu receptors for buprenorphine over other opioids, it ultimately is able to rapidly improve withdrawal symptoms and can eliminate the need for long-term supportive care.

Several case reports have been published recently demonstrating the efficacy of buprenorphine in acute opioid withdrawal, but only in cases of mild or moderate withdrawal, with COWS scores at buprenorphine induction ranging from 4 to 17 [2–4, 8]. We have presented a unique case demonstrating that buprenorphine is efficacious for significant reduction of withdrawal symptoms in moderately severe acute precipitated opioid withdrawal.

## Data Availability

Not applicable.
